# Doxycycline for transgene control disrupts gut microbiome diversity without compromising acute neuroinflammatory response

**DOI:** 10.1186/s12974-023-03004-4

**Published:** 2024-01-04

**Authors:** Emily J. Koller, Caleb A. Wood, Zoe Lai, Ella Borgenheimer, Kristi L. Hoffman, Joanna L. Jankowsky

**Affiliations:** 1 Department of Neuroscience, Baylor College of Medicine, One Baylor Plaza, Mail Stop BCM295, Houston, TX 77030 USA; 2 Alkek Center for Metagenomics and Microbiome Research, Department of Molecular Virology and Microbiology, Baylor College of Medicine, Houston, TX 77030 USA; 3 Departments of Neurology, Neurosurgery, and Molecular and Cellular Biology, Huffington Center On Aging, Baylor College of Medicine, Houston, TX 77030 USA

**Keywords:** Doxycycline, Tetracycline transactivator, Gut microbiome, Antibiotic, Neuroinflammation, Lipopolysaccharide, LPS, Amyloid, APP transgenic mouse, Transcriptome, Microglia

## Abstract

The tetracycline transactivator (tTA) system provides controllable transgene expression through oral administration of the broad-spectrum antibiotic doxycycline. Antibiotic treatment for transgene control in mouse models of disease might have undesirable systemic effects resulting from changes in the gut microbiome. Here we assessed the impact of doxycycline on gut microbiome diversity in a tTA-controlled model of Alzheimer’s disease and then examined neuroimmune effects of these microbiome alterations following acute LPS challenge. We show that doxycycline decreased microbiome diversity in both transgenic and wild-type mice and that these changes persisted long after drug withdrawal. Despite the change in microbiome composition, doxycycline treatment had minimal effect on basal transcriptional signatures of inflammation the brain or on the neuroimmune response to LPS challenge. Our findings suggest that central neuroimmune responses may be less affected by doxycycline at doses needed for transgene control than by antibiotic cocktails at doses used for experimental microbiome disruption.

**Supplementary Information:**

The online version contains supplementary material available at 10.1186/s12974-023-03004-4.

## Introduction

The tetracycline transactivator (tTA) system is widely used for controllable transgene expression in mouse models of neurodegenerative disease [1–14]. In the tTA system, oral administration of tetracycline or its analog doxycycline (dox) is used to suppress transgene expression, or to activate transgenic expression with the reverse tTA system [15, 16]. While these systems provide a powerful and versatile platform for studying disease, off-target effects of dox have been noted [17]. Dox is used therapeutically to inhibit bacterial protein synthesis by interfering with ribosomal structure [18], however, it can also interfere with mitochondrial function and disrupt the gut microbiome [19–21].

The gut microbiome exists in a close relationship with the gastrointestinal tract to support metabolism, but also shapes nervous system development and disease through circulating metabolites [22–27]. Considerable attention has been focused on the role of gut microbiome in Alzheimer’s disease (AD) and Parkinson’s, where studies in both human cohorts and mouse models suggest a bidirectional relationship between gut bacteria and disease progression [23, 28–31]. Intentional disruption of the gut microbiome with high-dose antibiotic cocktail reduced neuropathology and altered microglial and astrocytic morphology in the brain [32–35]. Antibiotic-induced microbiome changes were reversed by fecal transplantation, and gut repopulation rescued effects in the brain [34, 36]. Subsequent work identified microglia as a central mediator of microbiome-associated changes in brain pathology [34, 37]. Together these studies showed that oral antibiotics can influence the progression and severity of neurodegenerative disease through their effect on the gut microbiome. These findings also raised concern that using dox to control transgene expression in tTA-dependent models of neurodegeneration might compromise the inflammatory responses needed to promote neuropathology.

Here we set out to determine whether dox treatment at doses used for transgene control altered the gut microbiome and whether any changes to bacterial populations would recover after dox withdraw. We also wanted to know if dox treatment—and its potential impact on the microbiome—would affect the neuroimmune response in the brain. Given the extensive literature describing a link between antibiotic-induced changes in the gut and amyloid pathology in the brain, we focused on a tTA-controlled model of Alzheimer’s amyloidosis [6]; however, we expect our findings will be broadly applicable to other tTA-dependent models of neurological disease.

## Methods

### Mice

CaMK2α-tTA mice (also known as CaMKIIα-tTA) were derived from the Jackson Laboratory strain #003010 (38. tetO-APPswe/ind line 102 mice were described by Jankowsky et al. {Jankowsky, 2005 #565) and are available as MMRRC stock #34845-JAX. Both lines were maintained by backcrossing on a C57BL/6 J background for more than 20 generations. tTA and APP lines were intercrossed to generate bigenic CaMKIIα-tTA; tetO-APP offspring. Males from this cross were mated with wild-type FVB/NJ females to produce tTA;APP double transgenic mice (hereafter referred to as APP/TTA or Tg) and wild-type (WT) siblings on an F1 FVBB6 background for this study. Animals of both sexes were used for all experiments; no animals were excluded from analysis. Animals were maintained on 5V5M breeder chow or 5V5R standard diet (LabDiet), with or without dox as noted below. Animals were raised in static isolator caging under 11:13 h lighting. All animal work was reviewed and approved by the Baylor College of Medicine Institutional Animal Care and Use Committee.

### Doxycycline treatment

Half of the animals studied here were treated with dox from postnatal day 3 until 6 weeks of age (*n* = 16, 10 WT and 6 Tg); the other half remained on standard unmedicated 5V5M and 5V5R chow throughout life (*n* = 20, 13 WT and 7 Tg). LabDiet 5V5M breeder chow was formulated with 100 mg/kg doxycycline (dox, BioServ #F10089); standard chow consisted of 5V5R diet formulated with 100 mg/kg dox (BioServ #F10088). Dox-medicated breeder chow was administered to dams and litters beginning on postnatal day 3 (P3) and continued until weaning at postnatal day 21 (P21). Weanlings were maintained on dox-medicated 5V5R chow until 6 wk of age (P42), and then switched to non-medicated 5V5R diet until harvest at 12 wk of age. Dox chow was changed wkly to prevent degradation of the antibiotic.

### Stool collection

Mice were placed in clean, empty, autoclaved cages and allowed to explore until they defecated. Stool was retrieved and snap-frozen in sterile tubes on dry ice for later analysis. Stool was collected at 6 wk of age immediately prior to ending dox treatment, and again at 12 wk of age following 6 wk of dox washout. Samples were stored at − 80 until they could be processed together as a complete batch.

### LPS treatment

Following the final stool collection at 12 wk of age, a subset of the WT mice was systemically injected i.p. with 2 mg/kg lipopolysaccharide dissolved in sterile saline (LPS, Sigma-Aldrich, #L3024-5MG). Control mice were injected with saline, these saline-injected WT mice had not been sampled for microbiome analysis. Mice were harvested 18 h after injection.

### Tissue harvest

All animals were harvested at 12 wk of age. Mice were killed by pentobarbital overdose and transcardially perfused with PBS. Brains were removed and hemisected along the midline. One hemisphere was post-fixed in 4% paraformaldehyde overnight at 4 °C; the other hemisphere was sub-dissected to isolate the cortex which was snap-frozen on dry ice and stored at − 80 °C. The following day, the fixed hemisphere was transferred to 30% sucrose in PBS, kept at 4 °C until equilibrated, and then stored at − 80 °C. Brains were sagittally sectioned with a freezing sliding microtome at 35 um; sections were stored in cryoprotectant at − 20 °C.

### Final group sizes and sex distributions

**Table:** 

Genotype	Age	Chow	LPS/saline	Sample sizes					
				Microbiome		Microbiome/NanoString		NanoString	
				M	F	M	F	M	F
WT	6 wk / 12 wk	Unmedicated	LPS	–	–	3	2	–	–
WT	6 wk / 12 wk	Dox	LPS	–	–	3	3	–	–
Tg	6 wk / 12 wk	Unmedicated	–	3	4	–	–	–	–
Tg	6 wk / 12 wk	Dox	–	3	3	–	–	–	–
WT	12 wk	Unmedicated	Saline	–	–	–	–	6	2
WT	12 wk	Dox	Saline	–	–	–	–	1	3

### 16S sequencing and analysis

Total genomic DNA was extracted from stool samples using the DNeasy PowerSoil Pro Kit (Qiagen). Sequencing libraries were generated via PCR, using primers targeting the V4 region of the 16S rRNA gene (forward: GTGCCAGCMGCCGCGGTAA; reverse: GGACTACHVGGGTWTCTAAT). Primers used for amplification also contained adapters for sequencing, and a single-index barcode was included on the reverse primer. Libraries were sequenced on the MiSeq platform (Illumina) using the 2 × 250 bp paired-end protocol. Raw sequencing files were converted to FASTQ and demultiplexed using ‘bcl2fastq’ software (Illumina). Demultiplexed reads were quality filtered and merged before clustering into operational taxonomic units (OTUs) at similarity cutoff 97% using the UPARSE algorithm [24]. Taxonomy was determined by mapping OTUs to a V4 region-limited version of the SILVA database (v.138.1) [25]. Prior to analysis, read counts were rarefied to 16,950 reads/sample, a depth that enabled sufficient saturation of bacterial richness. Microbiome analysis was performed using Agile Toolkit for Incisive Microbial Analyses (ATIMA) (atima.research.bcm.edu). Both α- and β-diversity were calculated using OTU level taxa. For evaluations of α-diversity and taxa abundance, Kruskal–Wallis and Mann–Whitney statistical tests were employed. Statistical assessment of β-diversity was determined using permutational multivariate ANOVA (PERMANOVA). Raw FASTQ files and sample metadata for 16S sequencing were deposited at the Sequence Read Archive (SRA) (https://www.ncbi.nlm.nih.gov/sra) under BioProject PRJNA1017521.

### mRNA extraction

Brains were homogenized using the Bead Ruptor Elite Bead Mill Homogenizer (#19-042E, OMNI International) in homogenization buffer from the RNeasy Plus Micro Kit (#74034, Qiagen) with Reagent DX added at 1:200 dilution (#1011008, Qiagen) to reduce foaming. Total RNA was extracted from frozen cortical tissue using RNeasy Plus Micro Kit following the manufacturer’s instructions. RNA quality and concentration were measured using a NanoDrop UV–vis spectrophotometer (#13-400-519, ThermoFisher).

### NanoString nCounter assay

RNA samples were hybridized with the NanoString nCounter Mouse Neuroinflammation Panel and processed by the NanoString Technologies Proof-of-Principle laboratory (Seattle, WA). The resulting data were exported and provided as a CSV file.

### NanoString data analysis

Differential gene expression analysis was performed using the edgeR pipeline v3.38.4 [26]. Counts were then normalized to library size using the ‘Upper Quartile’ method. Principal component analysis was first performed on normalized expression data to identify potential outlier samples. No samples were removed from downstream analysis. Counts were first normalized to remove variation using positive and negative control genes in the RUVg function within RUVSeq v1.30.0 [27]. Results were calculated via the “likelihood ratio testing” parameter and Benjamin–Hochberg p-adjustment method. Genes with adjusted *p*-value < 0.05 and absolute value of log_2_FC above 0.5 were considered statistically significant. Heatmaps was created using ‘pretty heatmap’ package in R v1.0.12. Heatmap for all DEGs was generated using log_2_-transformed counts and scaled by row where colors represent row z-scores. Heatmap to compare between dox + LPS vs untreated and LPS vs untreated was generated using log_2_FC (fold change) values; colors represent raw log_2_FC values. Volcano plots were generated using the ‘Enhanced Volcano’ package in R v1.14.0 [28]. All genes were included in the analysis. Raw and processed NanoString data were deposited at Gene Expression Omnibus (GEO) (http://www.ncbi.nlm.nih.gov/geo/) under accession ID GSE236242.

### Immunohistochemistry

Brain sections were removed from cryoprotectant, washed in TBS, and treated with 0.9% hydrogen peroxide in TBS-T (1 × TBS + 0.01% Triton X-100) for 30 min at room temperature (RT). After washing with TBS again, sections were blocked for 2 h at RT with 5% goat serum in TBS-T. Sections were then incubated overnight at 4 °C in primary antibody diluted 1:1000 in blocking solution (GFAP, # Z033429-2 Agilent Technologies/Dako; Iba1, #019-19741, Wako). The next day, sections were washed with TBS, and incubated in biotinylated secondary antibody diluted 1:1000 in blocking solution for 2 h at RT (Vectastain Elite ABC Kit, Peroxidase Rabbit IgG: #PK-6101, Vector Laboratories). Sections were washed again before being incubated in A + B solution diluted in TBS (50 μl reagent A + 50 μl reagent B per 10 ml TBS) for 30 min at RT. After washing, antibody binding was detected with DAB Substrate Kit, Peroxidase (#SK-4100, Vector Laboratories). Sections were mounted onto Superfrost Plus slides (#12-550-15, Fisher Scientific), dried overnight, dehydrated through ethanol into xylene, and cover-slipped with Permount (#SP15-100, Fisher Scientific). Brightfield images were captured at 40 × magnification using a Zeiss AxioImager Z.2 microscope (Carl Zeiss AG, Oberkochen, Germany). Exposure time and lamp intensity were constant for all sections.

### Immunofluorescence

Brain sections were removed from cryoprotectant, washed in TBS, and blocked for 1 h at RT with 5% goat serum in TBS-T. Sections were then incubated overnight at 4 °C in primary antibody diluted in blocking solution (Ab9, 2.4 μg/ml, [39]). The next day, sections were washed with TBS and incubated in secondary antibody diluted 1:500 in blocking solution for 2 h at RT (Gt anti-Ms IgG2a-568, #A21134, Invitrogen). Sections were washed again before being incubated for 8 min at RT in 0.002% thioflavin-S diluted in TBS. Sections were then quickly rinsed twice in 50% ethanol, washed in TBS, mounted onto Superfrost Plus slides (#12-550-15, Fisher Scientific) and cover-slipped with Fluoromount-G (#0100-01, Southern Biotech). Fluorescent images were captured at 5 × magnification using a Zeiss AxioImager Z.2 microscope.

## Results

### Chronic dox administration perturbs the gut microbiome

We set out to test whether dox treatment used to control transgene expression in our APP/TTA model of Alzheimer’s disease might affect brain phenotypes by altering the gut microbiome. We also wanted to know if any change to the gut microbiome caused by dox exposure could be reversed by an equal period of drug washout. Our experimental design thus set out to answer three main questions: 1. How does dox treatment affect the gut microbiome composition? 2. Can normal microbiome composition be restored by drug withdrawal? 3. Does prior dox exposure affect the CNS response to immune challenge?

**Fig. 1:**
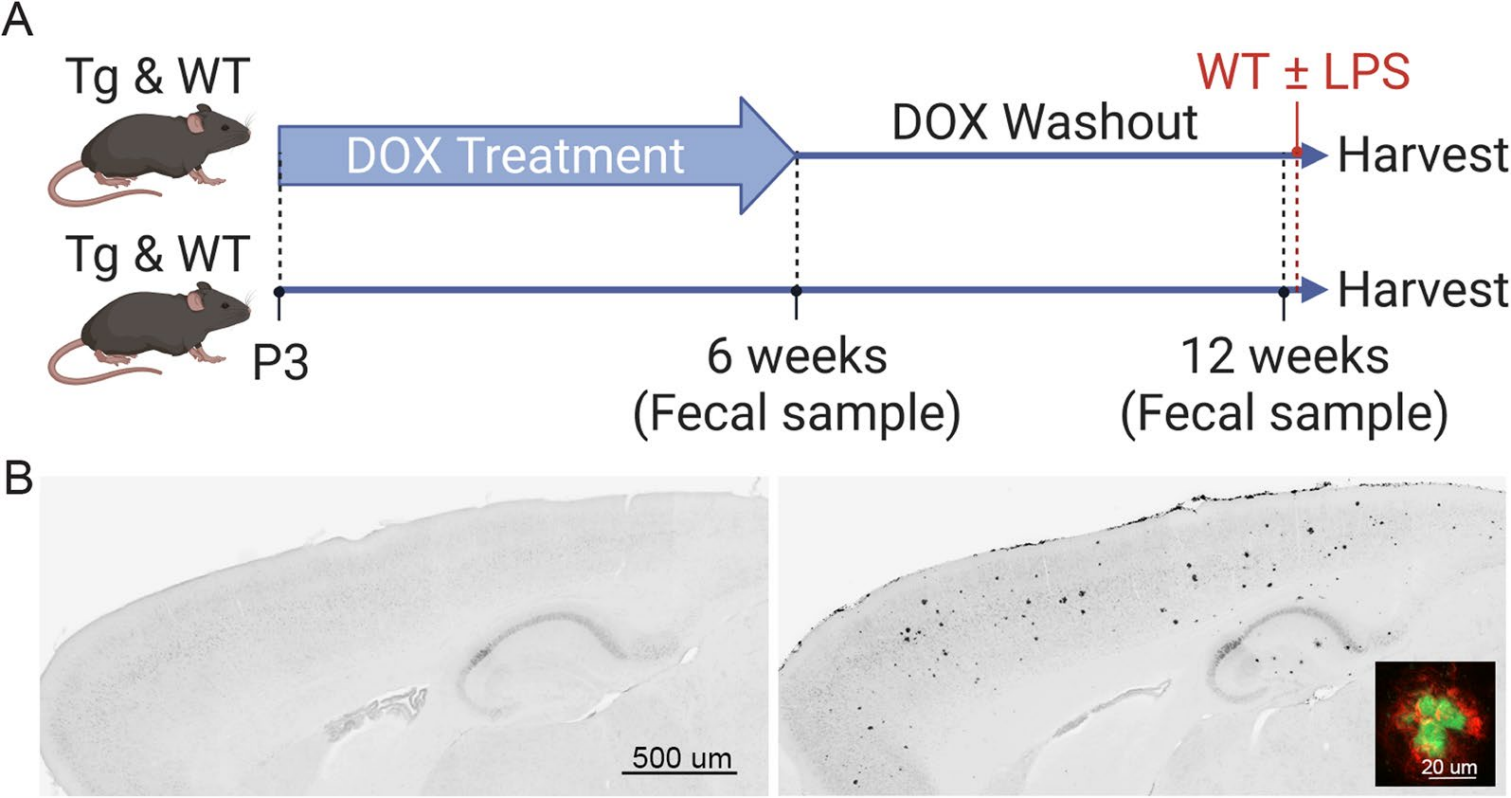
Schematic of experimental design.** A** Transgenic APP/TTA (Tg) and wild-type (WT) mice were split into two groups: one group received dox chow from P3 until P42, the other group received standard chow. At 6 wk, a fecal sample was collected from all mice and dox chow was removed. At 12 wk, another fecal sample was collected from all mice. Following fecal sampling at 12 wk, WT mice were used for LPS injection. Additional WT mice were injected with saline at 12 wk as controls for LPS without prior microbiome sampling. All mice, Tg and WT, were harvested 18 h after injection of the WT mice. **B** Aβ immunostaining of Tg mice harvested at 12 wk. Mice treated with dox for 6 wk followed by 6 wk of transgene expression showed no evidence of amyloid pathology (*left*). Amyloid pathology was observed across the cortex and hippocampus in untreated mice that expressed transgenic APP from birth (*right*). Most plaques were fibrillar deposits and co-labeled for Aβ (*red, inset*) and thioflavin-S (*green*). Created with BioRender

The current study was based on past work in which we treated APP/TTA mice (Tg) with dox for the first 6 wk of life to prevent transgene expression during postnatal brain development. This treatment period diminished hyperactivity and epileptiform activity in the adult that can interfere with cognitive testing [40, 41]. We administered dox from postnatal day 3 (P3) until 6 wk of age (P42); dox was then removed from 6 to 12 wk of age. Additional control animals were left untreated; both APP/TTA and WT mice were tested in each group. Fecal samples were collected at 6 and 12 wk to assess microbiome composition during dox treatment and following dox withdraw. One day prior to harvest, half of the WT mice received a single i.p. LPS injection to test whether prior dox exposure altered the neuroinflammatory response to an acute systemic challenge (Fig. 1A). By 12 wk of age, untreated Tg mice had developed a moderate plaque load across the cortex and hippocampus, while mice treated with dox for 6 wk had no amyloid pathology (Fig. 1B).

**Fig. 2:**
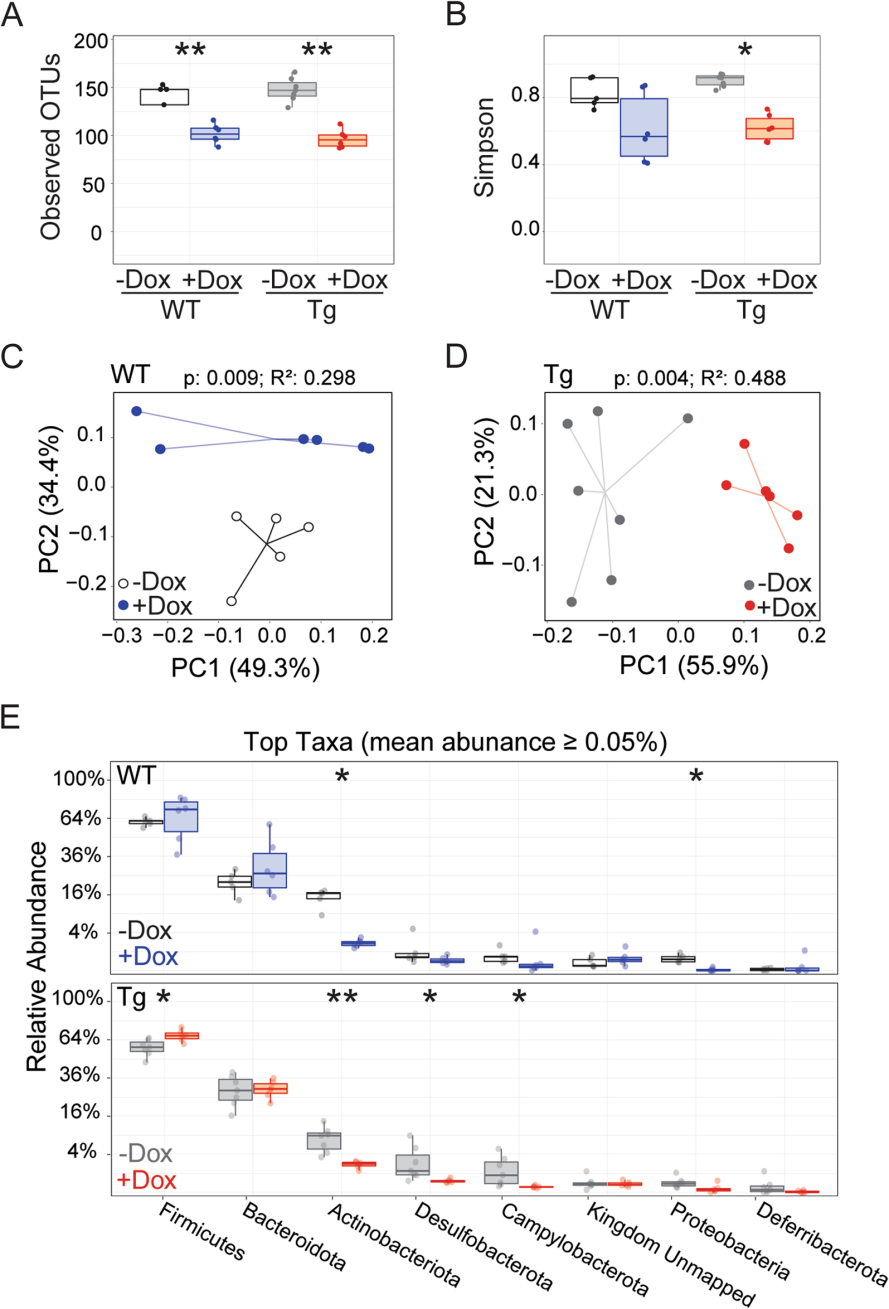
Dox treatment disrupts the gut microbiome in both Tg and WT mice. Analysis of gut microbiome from stool samples collected at 6 wk of age while half of each genotype was still receiving dox chow, focusing on the effect of dox exposure. **A, B** Observed OTUs (**A**) and Simpson index (**B**) reveal that dox treatment reduced α-diversity in both Tg and WT mice. **C, D** Principal coordinate analysis (PCoA) of weighted UniFrac distances indicate that dox altered β-diversity in both WT (**C**) and Tg mice (**D**). **E** The relative abundance of bacterial taxa was shifted by dox treatment in both genotypes. Statistical testing: Kruskal–Wallis (**A**, **B**), PERMANOVA (**C**, **D**), and Mann–Whitney U, reporting FDR-adjusted *p*-value (**E**). *n* = 5–7 mice/group. *p > 0.05, **p > 0.01. *Red* and *blue* = dox-treated Tg and WT, respectively; g*rey* and *white* = untreated Tg and WT, respectively

We first assessed whether 6 wk of dox treatment affected composition of the gut microbiome in each genotype (Fig. 2). 16S rRNA sequencing revealed that dox exposure decreased the number of bacterial operational taxonomic units (OTUs), a proxy for species, by a similar degree in both Tg and WT mice (Fig. 2A). In addition to decreasing the diversity of bacteria present, dox treatment reduced the evenness of microbial distribution across species in Tg mice, measured by Simpson index for α-diversity. WT mice treated with dox showed a trend towards lower evenness, but this change did not reach significance (Fig. 2B). Dox treatment also diminished the overlap in microbial community structure with untreated controls for both genotypes, measured by the weighted UniFrac metric for β-diversity (Fig. 2C, D). Finally, we found that dox affected distinct microbial phyla in each genotype. Firmicutes, Desulfobacterota, and Campylobacterota were reduced in only Tg animals, while Proteobacteria were significantly reduced in only WT mice. Actinobacteriota was the only abundant species (> 0.05%) that was significantly reduced in both genotypes (Fig. 2E). Taken together, our results indicate that 6 wk of dox treatment significantly diminished the number of different microbial phyla in both genotypes, but influenced bacterial evenness and composition in slightly different ways for Tg and WT animals.

**Fig. 3:**
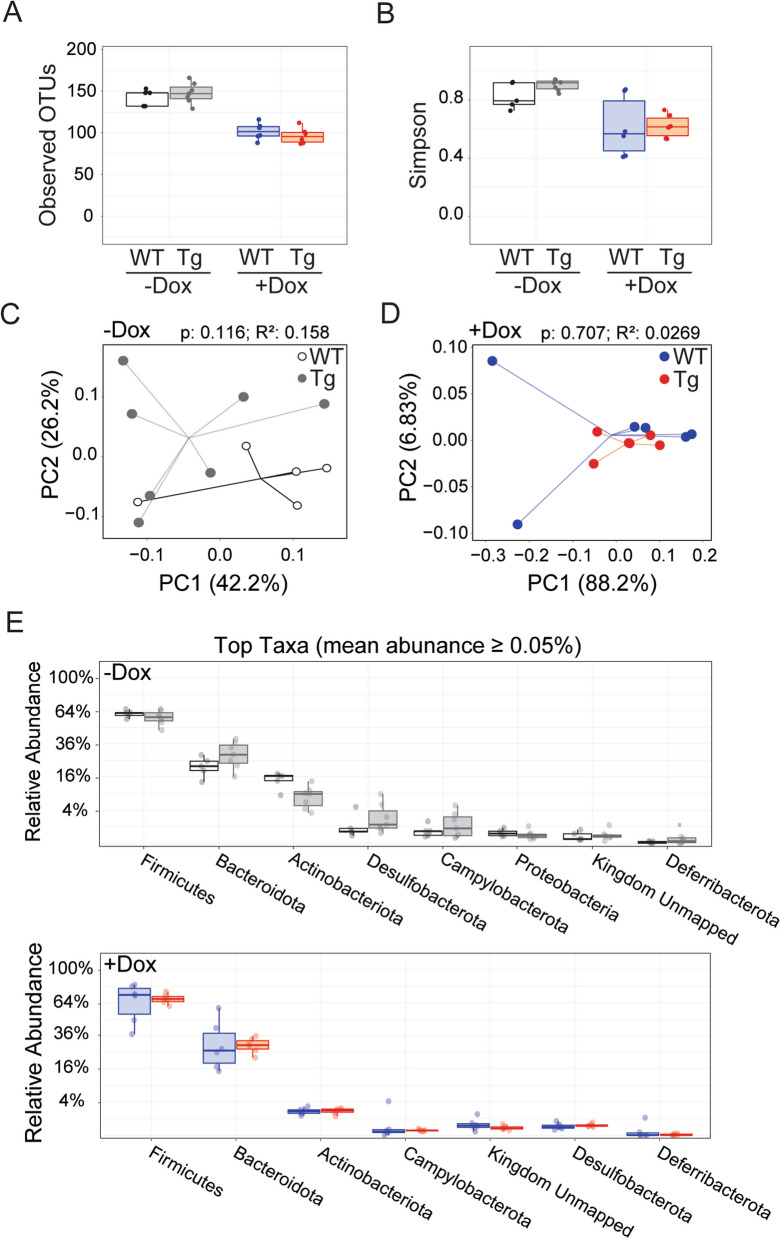
APP overexpression does not affect gut microbiome diversity prior to amyloid onset. Analysis of gut microbiome from stool samples collected at 6 wk of age while half of each genotype was still receiving dox chow, focusing on the effect of genotype. **A, B** Graphs of observed OTUs (**A**) and Simpson index (**B**) reveal that genotype had no effect on α-diversity at this age. Both measures were similar across genotypes whether APP overexpression was active (in untreated Tg mice) or not (with dox suppression). **C, D** PCoA plots of weighted UniFrac distances indicate that genotype did not affect β-diversity in untreated (**C**) or dox-treated mice (**D**). **E** The relative abundance of bacterial taxa is similar between genotypes at 6 wk of age for both treatment conditions. Statistical testing: Kruskal–Wallis (**A, B**), PERMANOVA (**C, D**), and Mann–Whitney U (E). n = 5–7 mice/group. *Red* and *blue* = dox-treated Tg and WT, respectively; *grey* and *white* = untreated Tg and WT, respectively

We next assessed whether genotype affected gut microbial composition in the absence of dox. Untreated Tg mice begin to accumulate Aβ by 6 wk of age, although this is prior to overt amyloid deposits. We also compared the effect of genotype on the microbiome response to dox. Results of these experiments were easy to summarize: we found no difference between genotypes for any measure of microbial diversity at 6 wk of age. OTU and Simpson index measures of α-diversity were similar between WT and Tg mice in untreated mice (Fig. 3A, B); both OTU and Simpson decreased with dox treatment, but did so equally in both genotypes. The weighted UniFrac measure of β-diversity was also similar between genotypes for untreated mice and remained so with dox treatment (Fig. 3C, D). Finally, relative abundance of the most prevalent taxa was similar between genotypes for each treatment condition (Fig. 3E). These results suggest that genotype had little effect on gut microbiome composition at 6 wk of age, regardless of whether APP overexpression was active or not.

### Changes in the gut microbiome persist after dox washout

**Fig. 4:**
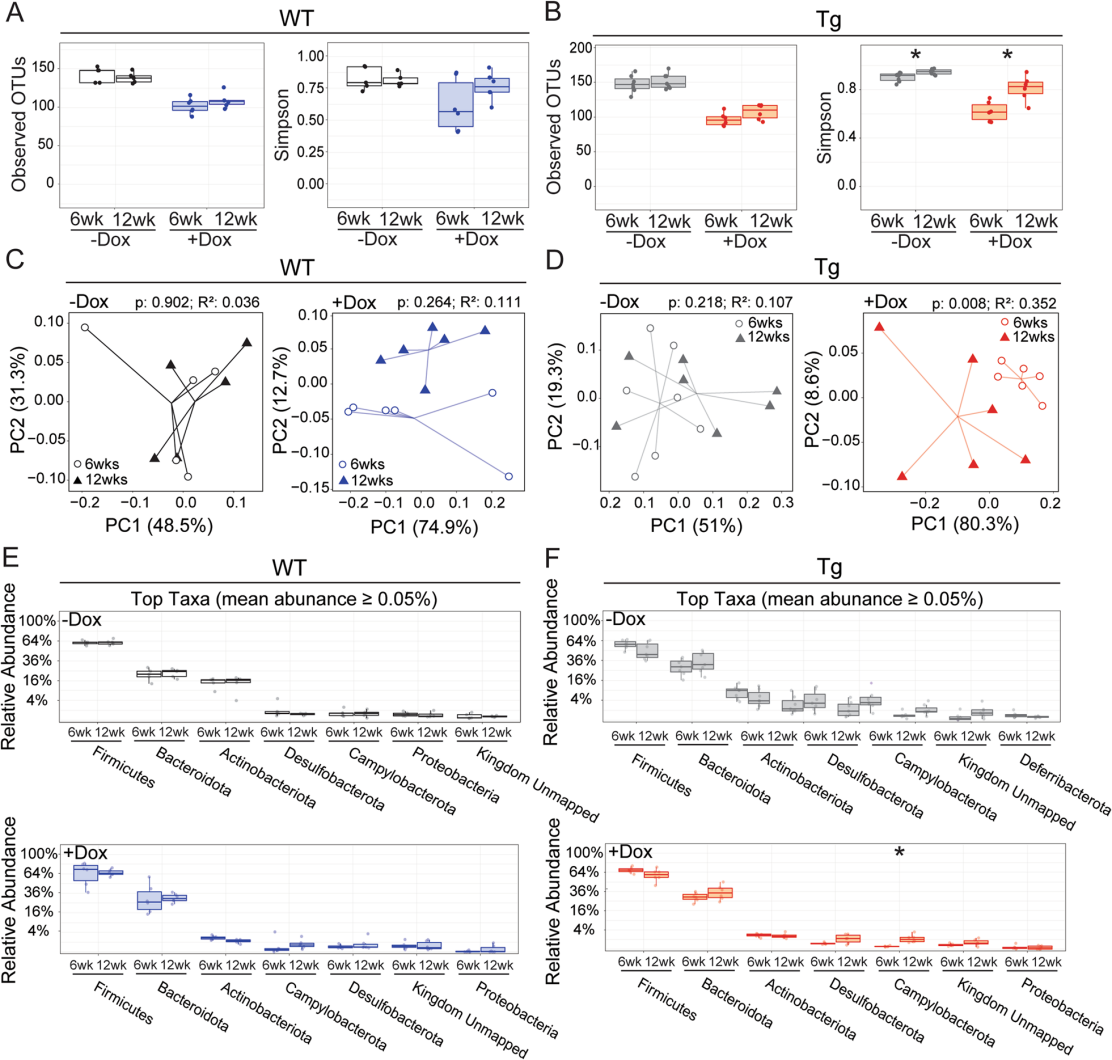
Changes to the gut microbiome largely persist following dox washout. Analysis of gut microbiome from stool samples collected at 6 and 12 wk of age, assessing the effect of time in untreated mice and of drug washout in dox-treated mice. **A, B** Observed OTUs (left) and Simpson index (right) as a function of age in untreated mice and of drug washout in dox-treated mice. Observed OTUs are unaffected by time and washout, while Simpson is increased in Tg mice both with age and drug removal. (**A**, WT; **B**, Tg). **C, D** PCoA plots of weighted UniFrac distances show that the overlap in species (β-diversity) was changed by drug washout for Tg mice but not WT, while neither genotype is altered by age alone (**C**, WT; **D**, Tg). **E**, **F** Relative abundance of bacterial taxa was unchanged by age alone (limited to taxa with average abundance ≥ 0.05% across all samples); only one phylum increased significantly upon drug washout in Tg mice (**E**, WT; **F**, Tg). Statistical testing: Kruskal–Wallis (**A, B**), PERMANOVA (**C, D**), and Mann–Whitney U, reporting FDR-adjusted *p*-value (**E, F**). *n* = 5–7 mice/group. *p < 0.05. *Red* and *blue* = dox-treated Tg and WT, respectively; *grey* and *white* = untreated Tg and WT, respectively

We next examined the gut microbiome at 12 wk of age, following 6 wk of dox washout, to determine whether any changes caused by prior dox treatment could be recovered upon drug withdrawal (Fig. 4). Alongside this comparison, we also evaluated the effect of age on untreated WT and Tg mice to ensure that any changes due to dox washout were not simply explained by time.

On its own, age had a small impact on Simson index in Tg mice, but no impact on any other measure we tested. This allowed us to focus on changes due to dox withdraw in both genotypes. We found that reduction in the number of bacterial species present (OTUs) caused by 6 wk of dox treatment persisted at 12 wk in both genotypes, long after dox was cleared from the system (Fig. 4A, B). Simpson index increased after dox washout, suggestive of recovery, but reached significance only for Tg mice (Fig. 4B). Changes in β-diversity supported this finding (Fig. 4C, D). While the principal coordinate analysis (PCoA) of weighted UniFrac distances hinted at growing separation between the microbiomes of dox-treated WT mice at 6 and 12 wk, this shift was only significant with dox washout for Tg mice (Fig. 4D). Finally, the relative abundance of the most common phyla remained similar following dox washout, with only Campylobacterota increasing significantly in Tg mice (Fig. 4E, F). Taken together, the dramatic suppression of gut microbial diversity caused by 6 wk of dox administration showed little recovery after 6 wk of drug washout.

**Fig. 5:**
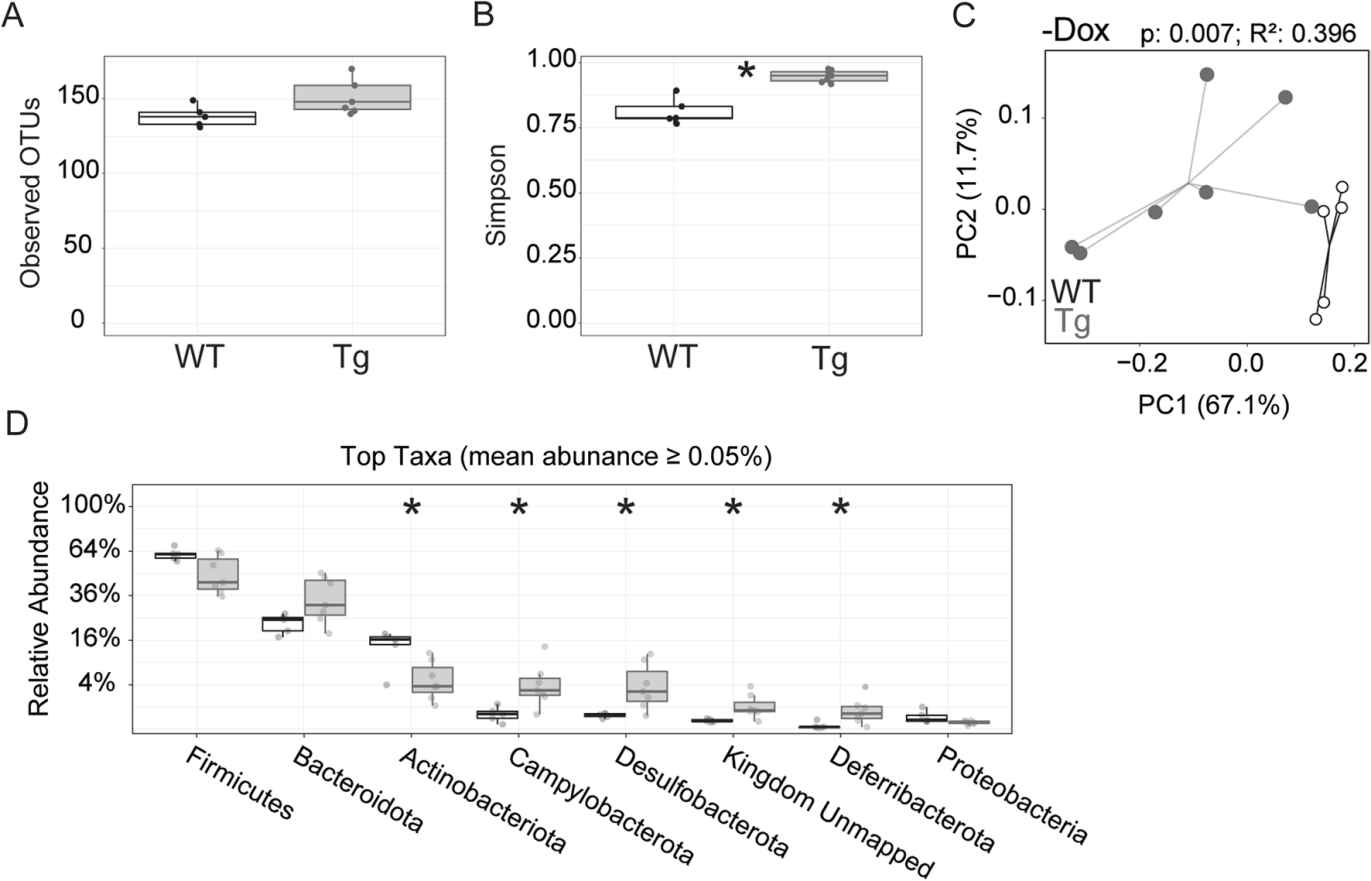
APP overexpression alters the gut microbiome at 12 wk of age with the onset of amyloid pathology. Analysis of gut microbiome from stool samples collected at 12 wk of age, assessing the effect of genotype in untreated mice. **A, B** Observed OTUs (**A**) and Simpson index (**B**) reveal that the onset of amyloid formation in untreated Tg mice significantly increased bacterial evenness (α-diversity) at this age. **C** PCoA plots of weighted UniFrac distances indicate that genotype significantly altered β-diversity. **D** The relative abundance for 5 of the top 8 bacterial taxa was significantly altered in Tg mice with the onset of amyloid deposits. Statistical testing: Mann–Whitney (**A, B**), PERMANOVA (**C, D**), and Mann–Whitney U (**E**). *n* = 5–7 mice/group. *p < 0.05. *Grey* and *white* = untreated Tg and WT, respectively

Finally, we examined whether the onset of amyloid plaques influenced microbiome diversity. By 12 wk of age, untreated Tg mice would harbor both high levels of soluble Aβ and have formed initial insoluble amyloid deposits. APP overexpression by itself was not sufficient to affect bacterial diversity at 6 wk of age (Fig. 3), but by 12 wk of age both α- and β-diversity differed significantly between Tg and WT mice (Fig. 5). Outcomes were similar for both sexes within each genotype, although sample sizes were not powered for sex as an additional variable. The number of bacterial species measured by sequencing did not differ between genotypes (observed OTUs), but Tg mice had higher Simpson values than WT controls (Fig. 5A, B), suggesting that amyloid accumulation in the brain increased variation between microbial populations in the gut. The onset of plaques in Tg mice diminished the overlap in microbial species between genotypes, with significant separation of the two genotypes on PCoA plot of weighted UniFrac values (Fig. 5C). In addition, the relative abundance of multiple major bacterial phyla shifted in Tg mice compared to WT siblings (Fig. 5D). Actinobacteriota were decreased in abundance, offset by increases in Campylobacterota, Desulfobacterota, and Deferribacterota. Remarkably, dox exposure abolished all of these genotype effects (Fig. 4 and data not shown). Our results are consistent with past work showing that the onset of amyloid formation in the brain of transgenic APP mice can influence the gut microbiome of untreated mice, but that antibiotic treatment trumps genotype in shaping microbial survival [28].

As one last examination of the data, we tested for the effects of genotype, treatment, age, and sex in a combined analysis that included all of our microbiome samples. This analysis revealed that dox treatment had the greatest impact on β diversity as measured by weighted unifrac, accounting for 18.8% of the total variance across samples (*p* < 0.001). Age was also a significant contributor to β diversity, accounting for 6.3% of the variance (*p* < 0.05). Neither sex nor genotype were significant factors in overall variance.

### Neuroinflammatory response to LPS challenge is not altered by dox-induced changes in the gut microbiome

Past studies have shown that antibiotic treatments sufficient to alter the gut microbiome also affected neuroinflammatory responses in the brain [28, 42]. This consequence of gut microbiome disruption could confound experiments using dox-controllable models of neurodegenerative disease such as ours, especially if the consequences of acute dox treatment persisted through the washout period. While prior work examined how disruption of the gut microbiome with antibiotic cocktail influenced the microglial reaction to amyloid plaques, here we opted for a more acute readout of whether persistent microbiome changes due to our single antibiotic regimen would impact neuroinflammation. We focused on WT mice to avoid the confound of amyloid status in Tg mice, and used a single systemic injection of bacterial lipopolysaccharide (LPS) to evoke neuroinflammation over a short and well-documented time course. We divided mice into 4 treatment groups: half the mice were treated with dox from P3–P42, the other half were left untreated. Half of each dox condition was challenged with LPS, the other half was injected with saline. We used transcriptional profiling of cortical tissue to gain a broad view of LPS-induced changes in the brain and whether these were altered by prior dox treatment.

**Fig. 6:**
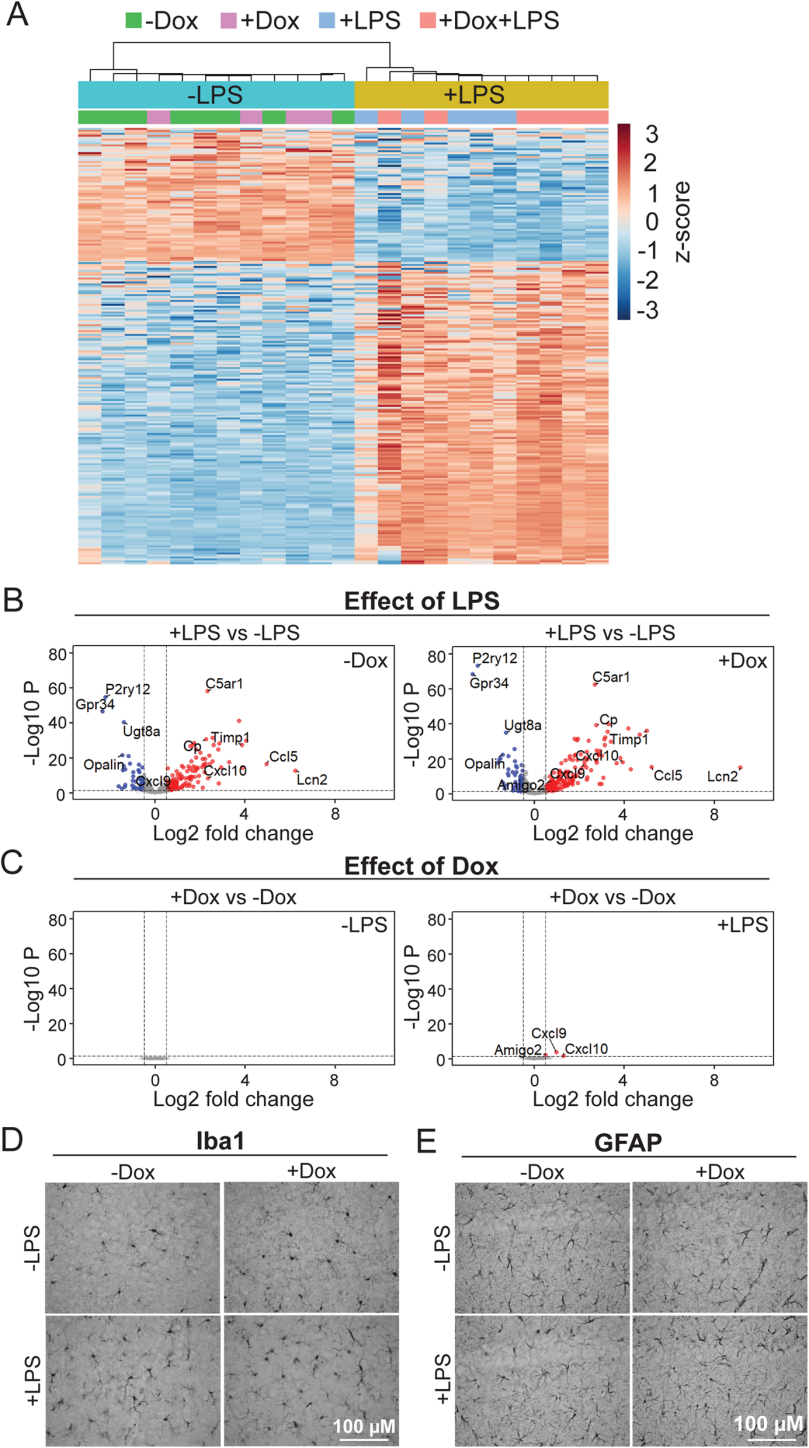
Transcriptional response to LPS challenge is unaffected by prior dox treatment.** A** Transcriptomic analysis of neuroinflammatory genes in cortical tissue of 12-wk-old WT mice challenged with systemic LPS or saline 18 h prior to harvest. Heatmap shows log-transformed counts of differentially expressed genes for each sample, colors represent row-normalized z score. Unbiased hierarchical clustering separated animals by LPS treatment, but not dox condition. **B** Volcano plots show that the same genes are up- or down-regulated by LPS challenge in both dox-treated and untreated mice. Genes with an adjusted *p*-value < 0.05 and an absolute value of logFC > 0.5 were considered statistically significant and are indicated with red (upregulated) and blue (downregulated) dots. **C** Volcano plots show that few genes differ significantly as a function of dox treatment within LPS-challenged or saline-injected animals. **D, E** Representative 40 × images of Iba1 immunostaining in the cortex (**D**) and GFAP immunostaining in the hippocampus (**E**) of WT mice show little difference in morphology or distribution of either cell type between conditions. *n* = 5–7 per group. Scale bar: 100 µm

LPS challenge substantially altered a major fraction of genes included in the NanoString neuroinflammatory panel. In dox-untreated mice, LPS upregulated 120 genes and downregulated 48 within the 770 gene panel (false discovery rate < 0.05, Additional file 1: Table S1). In dox-treated mice, LPS affected a similar number of genes, upregulating 149 and downregulating 49. Over 90% of the differentially expressed genes (DEGs) from dox-treated mice overlapped and were concordant with DEGs from mice that had never been treated with dox. Unbiased hierarchical clustering of DEGs revealed that animals grouped solely by LPS condition; prior dox treatment had no effect on clustering (Fig. 6A). Dox-treated and untreated mice intermingled in the hierarchy, but samples clearly divided between LPS and saline conditions. The effect of sex was also minor compared to that of LPS treatment; principal component analysis demonstrated that LPS was responsible for 69.4% of the variance between samples while sex contributed just 6.1% (data not shown). LPS treatment caused consistent up- or down regulation of the same genes in dox-treated and untreated mice (Fig. 6B). In contrast, dox treatment had little effect on gene expression within LPS or saline conditions (Fig. 6C). This data suggests that prior dox exposure had no effect on basal expression of neuroinflammatory genes in the brain, and no appreciable effect on the transcriptional response to acute inflammatory challenge.

We next performed immunohistochemistry on the remaining brain tissue to visualize microglia using Iba1 and astrocytes using GFAP as the main responders to both acute and chronic inflammatory challenge. Despite the pronounced transcriptional changes induced by LPS, we found no noticeable differences in glial shape or distribution between conditions (Fig. 6D, E). Although the absence of a clear morphological response to LPS limits our interpretation, these findings are consistent with the conclusion that dox had no overt effect on basic phenotypes of these CNS cells.

## Discussion

We set out to test whether dox control of transgene expression in the CNS might unintentionally affect the gut microbiome. If so, would these effects persist after dox removal, and would prolonged changes in the gut microbiome affect neuroimmune responses in the brain? Past work had shown that high-dose antibiotic cocktails used to disrupt the gut microbiome affected immune responses in the brain, and that these changes in the gut altered progression of neuropathology in the brain associated with AD and other dementias [27, 28, 33, 34, 43]. Multiple tTA-based models for neurodegenerative disease have been produced, where unintended harm to the microbiome caused by dox exposure could influence experimental outcomes in the brain [1–14]. Here we show that although dox treatment caused persistent alterations in the gut microbiome, these changes had no overt effect on the brain’s transcriptional response to a widely used systemic immune challenge.

Two prior studies have examined gut microbial profiles following dox treatment in WT mice and both found that as little as 2 wk of oral dox administration significantly altered gut microbiome diversity and composition [19, 20]. Here we confirm and extend these findings by showing that the dox effect was similar but not identical in WT and Tg mice. The richness of bacterial communities was equally affected by dox exposure in both genotypes, but dox had a significant effect on microbial evenness only in Tg mice. Evenness in WT mice trended in the same direction as in Tg groups, suggesting that the genotype distinction might collapse with larger group sizes or longer treatment. More bacterial phyla were altered by dox in Tg mice than in WT, although again the trends were similar. These effects are consistent with the findings of Becker and Boynton [19, 20], with one notable exception. Our study found much smaller changes in the abundance of specific phyla following dox treatment than either of these two prior reports. These differences may be due to the dosing used for each study, which was 3–16 × higher in the prior studies but over a shorter duration than used here to control transgene expression. Taken together, these findings show that even low doses of dox given over several wks can disrupt multiple measures of gut microbiome stability.

Studies such as ours using the tet-off (tTA) system may use dox to withhold transgene expression for some period of time, but then usually release mice from dox to initiate the intended phenotype. This approach is used in many of our own studies to withhold APP overexpression until adulthood. Given this on-dox/off-dox experimental design, and knowing that even the low doses used to control transgene expression altered the gut microbiome, we next tested whether bacterial communities would recover following dox removal. Despite 6 wk of drug washout—allowing as much time off dox as the mice had spent on the drug—the microbiome still bore hallmarks of prior dox exposure. Some features did recover, such as the evenness of bacterial communities in Tg mice, while other features such as bacterial richness remained low in both genotypes. Again, these findings are consistent with prior work from Becker et al. who also saw recovery in bacterial evenness 4 wk after dox withdrawal [19]. The prolonged effect of prior dox exposure was not shared by another common antibiotic metronidazole, despite greater microbiome disruption during treatment [19]. We too were surprised by the persistent imprint dox had on microbial populations, although our work stopped short of testing longer washout periods required for the appearance of neuropathology in common tet-off transgenic models.

Changes in the gut microbiome have been increasingly shown to have a profound influence on brain development and function [27]. Complete loss of the gut microbiome by raising mice in germ-free conditions affects microglial development, maturation, and aging [26, 42, 44]. Induced gut dysbiosis using an antibiotic cocktail elicited more subtle changes in microglial structure but still had marked effects on neuroimmune response to systemic insult and CNS pathology [35, 45]. These findings were the main concern motivating our current study of dox treatment in the APP/TTA model. We opted to examine the impact of dox on glial reactivity using systemic LPS rather than amyloid formation for a faster readout, and focused on WT mice rather than Tg to avoid the offset in amyloid load that would arise from treating half our Tg mice with dox. We found that prior dox exposure had no effect on transcriptional signatures of neuroinflammation at baseline nor on the transcriptional response to LPS challenge. As expected, systemic LPS administration drove several hundred gene expression changes in the cortex, but did so equally in mice that had been treated with dox and those that had not. Despite marked changes in transcription, we saw no obvious effect of LPS on gross glial morphology in either dox condition. This was surprising, but we may simply have harvested the mice too soon after LPS challenge or used too little to see a reactive morphology. We also appreciate that our NanoString panel may have missed transcriptional changes that fell outside the 770 gene set. Taken together, our findings suggest that dox-induced changes in the gut microbiome did not overtly dampen brain’s response to immune challenge. Importantly, this finding is consistent with prior work by Dodiya et al. where single antibiotic treatment had little effect on amyloid progression in the brain [46].

The gut–brain axis exerts a bidirectional effect on both organs and so we additionally assessed the effect of APP overexpression on the gut microbiome [22, 27, 47]. We focused on untreated Tg mice to isolate the effect of genotype from that of dox exposure. Tg mice were initially no different than WT in bacterial richness and evenness, but by 12 wk these measures began to diverge. Age-dependent differences in the gut microbiome of other APP transgenic models have also been reported [48]. In two different APP/PS1 strains, the 5xFAD model and our APP/TTA mice here, microbiome properties first diverged from WT coincident with or shortly before the expected onset of amyloid pathology [28, 40, 49–51]. These findings suggest that neuropathology in the brain has systemic repercussions that measurably impact the gut microbiome across multiple models of Alzheimer’s amyloidosis.

Unlike many other APP transgenic models, female APP/TTA mice do not seem to show earlier pathology than males. Consistent with the apparent absence of sex bias in amyloid onset, we also found no significant differences in microbiome properties between males and females of either genotype. Few other studies have examined the microbiome of both sexes in amyloid mice, but where tested, gut dysbiosis was greater in male transgenic mice [1, 52]. Conversely, males are selectively sensitive to the effect of microbiome disruption on amyloid load in two separate APP/PS1 models [33, 36]. Although small group sizes in our study precluded rigorous testing of male vs. female outcomes, past work clearly demonstrates that sex and genotype can interact in similar amyloid models to shape gut–brain communication.

In summary, we show that dox treatment at doses commonly used for transgene control persistently disrupted the gut microbiome, yet dox-treated mice could still mount a robust inflammatory response to acute immune challenge. These results provide some reassurance that the use of dox for transgene control does not present a fatal flaw for studies where neuroinflammatory responses are an essential aspect of pathogenesis.

## Conclusion

We found that oral administration of doxycycline for transgene control in tTA-based models of neurological disease altered the gut microbiome, and that these changes in bacterial composition persisted long after drug withdrawal. While intentional disruption of the gut microbiome using antibiotic cocktails can suppress immune function in the brain, mice exposed to chronic dox treatment were able to mount a robust neuroinflammatory reaction to systemic LPS challenge. These findings allayed concern that dox treatment would impair neuroimmune responses as an unwanted artifact in studies of dox-controlled transgenic models of neurodegenerative disease. Controllable transgenic models offer a valuable tool for experimentally manipulating disease onset and region of interest, and the findings offered here provide a clearer understanding of their potential use and actual limitations.

### Supplementary Information

**Additional file 1.** Supplementary Table 1.

## Abbreviations

AD: Alzheimer’s disease
APP: Amyloid precursor protein
ATIMA: Agile Toolkit for Incisive Microbial Analyses
DEG: Differentially expressed gene
dox: Doxycycline
FC: Fold change
GFAP: Glial fibrillary acidic protein
Iba1: Ionized calcium-binding adaptor protein-1
OTUs: Operational taxonomic unit
i.p.: Intraperitoneal
LPS: Lipopolysaccharide
PBS: Phosphate buffered saline
PCoA: Principal coordinate analysis
RT: Room temperature
Tg: Transgenic
tTA: Tetracycline transactivator
TBS: Tris-buffered saline
wk: Weeks
WT: Wild-type
